# Application of Arsenic Trioxide-Based Combined Sequential Chemotherapy in Recurrent Resistant and Refractory Ovarian Cancers: A Single-Center, Open Phase II Clinical Study

**DOI:** 10.1155/2022/6243165

**Published:** 2022-08-31

**Authors:** Yingchao Yang, Xiaoping Li, Yue Wang, Xiaoyan Shen, Lijun Zhao, Yan Wu, Yi Li, Jianliu Wang, Lihui Wei

**Affiliations:** Department of Obstetrics and Gynecology, Peking University People's Hospital, No.11 Xizhimen South Street, Xicheng District, Beijing, China

## Abstract

**Objective:**

Arsenic trioxide (ATO) has been effectively used for the treatment of hematological malignancies and some solid tumors, while ATO effects were not tested clinically in epithelial ovarian cancer (EOC).

**Methods:**

Patients with primary or secondary platinum-resistant EOC were enrolled from October 2015 to December 2019. Patients were mainly treated with ATO-based combined sequential chemotherapy as follows: Regimen 1 (ATO combined taxanes weekly therapy); Regimen 2 (ATO + taxanes + 5-fluorouracil + adriamycin ± bevacizumab sequential chemotherapy), for 5 patients platinum-free interval >12 months, added oxaliplatin). Prespecified end points in this cohort included confirmed best overall response rate (ORR), disease control rate (DCR), progression-free survival (PFS), overall survival (OS), and safety.

**Results:**

A total of 33 patients were enrolled in this study. After a median follow-up time of 22.1 months (range 5.5–42.9 months), ORR was 42% and DCR was 85%. The overall PFS was 9.5 months (range 1–38.4 months). The main side effect was myelosuppression.

**Conclusions:**

ATO-based sequential combined chemotherapy is effective for primary and recurrent drug-resistant EOC patients in clinical phase II trials. The associated side effects could be controlled, while further study is needed.

## 1. Introduction

Platinum-based combined chemotherapy is an effective adjuvant treatment for epithelial ovarian cancer (EOC).

Primary systemic therapy regimens for EOC are paclitaxel combined carboplatin or carboplatin combined pegylated liposomal doxorubicin or docetaxel combined carboplatin. Bevacizumab is a vascular endothelial growth factor A (VEGF-A or VEGF) targeting monoclonal antibody and was the first approved angiogenesis inhibitor. The recently identified immune-modulatory roles of VEGF provide a powerful rationale for combination therapies. In which, bevacizumab combined chemotherapy or maintenance therapy followed by paclitaxel combined carboplatin, and bevacizumab is continued for up to 12 additional cycles or for up to 22 cycles.

However, after primary EOC treatment, 25% of patients develop resistance to chemotherapy, and 75% of EOC patients may relapse. Definition of platinum-sensitive versus platinum-resistant recurrence has been suggested that patients with a platinum-free interval (PFI) of six months or longer are considered to have “platinum-sensitive” malignancies, while those with a PFI of less than six months are considered to have “platinum-resistant” cancers, and the latter group includes women who experienced disease progression during first-line platinum-based therapy. The group is often referred to as having “platinum-refractory” disease [1], although some studies have challenged the relevance of the definition. Importantly, once drug resistance occurs, the lack of effective chemotherapy regimens seriously affects cancer survival and prognosis characteristics. Currently, the 2022 National Comprehensive Cancer Network (NCCN) Guidelines recommends cyclophosphamide (oral) ± bevacizumab, docetaxel, etoposide (oral), gemcitabine, liposomal doxorubicin ± bevacizumab, paclitaxel (weekly) ± bevacizumab, and topotecan ± bevacizumab for platinum-resistant EOC with poor prognosis [2]. Combining single-agent chemotherapy with or without bevacizumab, and polyADP ribose polymerase (PARP) inhibitors may be effective. Therefore, it is necessary to improve the efficacy of the existing and develop new treatment approaches in platinum-resistant cancer patients.

Arsenic trioxide (ATO) is a trivalent oxide of arsenic, which has unique antitumor effects and broad-spectrum activity. Several studies have reported that it was not only effective in the treatment of hematological malignancies but also in the treatment of some solid tumors, including esophageal, gastric, liver, and lung cancers [3]. ATO application as anticancer treatment in osteosarcoma patients at Bone Tumor Center of Peking University People's Hospital showed good short-term clinical effects [4]. We have recently reported the basic science and clinical application of ATO in resistant uterine malignancies [5], and the results showed that ATO was effective in patients with endometrial cancer [6, 7]. Currently, the anticancer effects of ATO are considered beneficial by gynecologic oncologists because of its low price, low side effects, and the wide range of antitumor effects. It has been suggested that ATO could be used for the treatment of recurrent and platinum-resistant ovarian cancer patients [8]. Accordingly, the purpose of this study was to study ATO-based combined sequential chemotherapy in the treatment of refractory and recurrent EOC, to evaluate its efficacy and safety classification of this approach, and to provide a new scheme recommendation for further clinical use of ATO in primary or secondary platinum-resistant EOC patients.

## 2. Methods

### 2.1. Clinical Data Collection

A total of 33 patients with recurrent/refractory EOC, primary fallopian tube cancer, or primary peritoneal cancer were enrolled in this study from October 2015 to December 2019. The study was approved by the ethics committee of People's Hospital Peking University. All enrolled patients signed the informed consent for the chemotherapy scheme.

### 2.2. Chemotherapy Regimen Schemes

All patients with platinum-resistant tumors were treated with combined sequential ATO-based chemotherapy. The scheme was as follows.

Regimen 1. ATO combined with taxanes weekly therapy. A chemotherapy regimen with 7 mg/m^2^ of ATO was given via intravenous drip QD for 8 d, and 80–100 mg/m^2^ of taxanes was given for day 1 and day 8, with a treatment interval of 3 weeks.

Regimen 2. ATO combined with multiple drugs with sequential combined chemotherapy. Chemotherapy regimen included 7 mg/m^2^ of ATO QD for 8 d; 80–100 mg/m^2^ of taxane intravenous drip, D1; 800–1000 mg/m^2^ of 5-fluorouracil intravenous drip, D3-D4; 40–50 mg/m^2^ of adriamycin intravenous drip, D8; and bevacizumab 7–10 mg/kg, D2, intravenous drip. For patients' platinum-free interval >12 months, added 110 mg/m2 of oxaliplatin, D8, intravenous drip, instead of adriamycin intravenous drip, D8.

The treatment was continued until the disease progressed or unacceptable side effect was observed. If unacceptable toxicity was directly caused by the study drug, the treatment was reduced by one dose grade or the drug administration was postponed appropriately. If the drug toxicity or study drug interruption lasted for 6 weeks after the last dose was administered, the study treatment was terminated.

### 2.3. Clinical Indicators and the Evaluation of Drug Efficiency

Targeted measurable lesions were assessed and documented before treatment. Tumor response was assessed according to Response Evaluation Criteria in Solid Tumors (RECIST) by clinicians using computed tomography (CT) scans, magnetic resonance imaging (MRI), or ultrasound examination after three and six cycles of ATO-based combined sequential chemotherapy [9]. The assessment was subsequently repeated every 3 months until confirmed disease progression or intolerability.

The primary assessment endpoint was the proportion of patients achieving an objective response according to RECIST version 1.1 [9]. The endpoint included patients who demonstrated complete or partial responses. Secondary assessment endpoints included progression-free survival (PFS), duration of response, disease control rate (DCR), and safety. PFS was defined as the interval from the start of the treatment to the day of disease progression, diagnosis, or death for any cause (which ever occurred first), or until the last PFS assessment for alive patients without cancer progression. Duration of the response was assessed in patients who achieved a response. The duration was defined as the time from the date of the first registered response until the date of documented cancer progression or death from any cause. Disease control rate (DCR) was defined as the proportion of patients who achieved a complete response (CR), a partial response (PR), or stable disease condition (SD). The objective response rate (ORR) was defined as the proportion of patients with PR or CR.

Tumor marker cancer antigen 125 (CA_125_) levels in blood plasma were determined before all treatments. The efficacy of CA_125_ was evaluated according to the modified international standard of Gynecologic Cancer Inter Group (GCIG) criteria [9]. Adverse events were assessed according to the common terminology criteria for adverse events defined by the National Cancer Institute [10].

### 2.4. Statistical Methods

We calculated the proportion of patients achieving responses and assessed 95% confidence intervals (CIs) using the Clopper–Pearson method and survival times and progression-free survival and associated 95% CIs using the Kaplan–Meier method. We used a log-rank test to compare the progression-free survival between patients with different grades of toxicity. The data of follow-up were collected by the cutoff date of March 10, 2022. SPSS (version 22.0) was used for analyses.

## 3. Results

### 3.1. Demographics, Clinical, and Baseline of EOC Characteristics

The general clinical and pathological characteristics, demographics, and baseline disease characteristics of 33 patients are shown in Table 1. The median age was 57 years (range: 37–69 years). The histopathological types were represented as follows: 27 cases of serous adenocarcinoma, 5 cases of clear cell carcinoma, and 1 case of serous endometrioid carcinoma. According to the 2018 FIGO (International Federation of Gynecology and Obstetrics) staging guidelines, there were 6 cases at stage IC-II, 18 cases at stage III, and 9 cases at stage IV. Previous chemotherapy regimens for relapsed drug-resistant patients were described as follows: first-line chemotherapy in 4 patients, second-line chemotherapy in 14 patients, and third-line chemotherapy in 15 patients. Tumor recurrence was observed at the following sites: 4 cases of liver metastasis, 3 cases of lung metastasis, 2 cases of bone metastasis, and 10 cases of multiple lymph node metastasis (including neck, mediastinum, and abdominal paraaortic lymph nodes). Among them, 88% (29/33 cases) received multiline chemotherapy, with 15 cases (45.45%, 15/33) with primary and 18 cases (54.55%, 18/33) with secondary drug resistance (see Table 1).

### 3.2. ATO-Based Sequential Combined Chemotherapy Regimen and Clinical Treatment

All 33 patients treated with ATO-based sequential combined chemotherapy regimen are shown in Table 2: Regimen 1 was administered in 6 patients; Regimen 2 was administered in 27 patients; for 5 cases platinum-free interval >12 months, added oxaliplatin). The patients received a median of 7 courses of chemotherapy (range: 3–12 cycles).

### 3.3. Efficacy of ATO-Based Sequential Combined Chemotherapy

**Figure 1:**
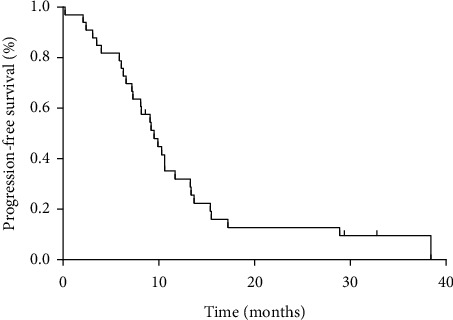
Kaplan–Meier graph for progression-free survival in patients.

**Figure 2:**
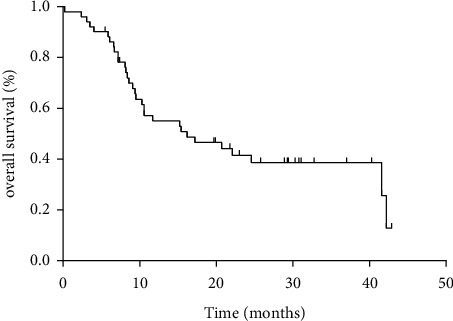
Kaplan–Meier graph for overall survival in patients.

The follow-up observation ended on March 10, 2022, with a median follow-up time of 22.1 months (range 5.5–42.9 months). Overall evaluation of 33 patients as the following results: it has been found that 6, 8, 14, and 5 patients (18%, 24%, 42%, and 15%) were in CR, PR, SD, and PD, respectively. ORR was 42% (14/33) and DCR was 84% (28/33), which are shown in Table 3. The median PFS time was 9.5 months (range 1–38.4 months) for 33 patients (Figure 1). By the follow-up deadline, 26 cases (26/33, 79%) had stable disease, and 24 cases (24/33, 73%) had OS ≥ 12 months. According to the survival curve, the median OS time was 16.2 months (Figure 2).

### 3.4. Major ATO Chemotherapy-Associated Common Side Effects

The most common adverse event (Table 4) was myelosuppression, which was defined as drug-related in all 33 cases (100%, 33/33). The grade 3 and above side effects included leukopenia, anemia and platelet decline, liver insufficiency, nausea, vomiting, constipation, and drug-induced liver damage.

## 4. Discussion

### 4.1. The Beneficial Effect of ATO-Based Sequential Combination Chemotherapy

Currently, the prognoses of primary and secondary platinum-resistant recurrent ovarian cancer are poor. NCCN recommended a variety of single-drug chemotherapy regimens, including paclitaxel weekly therapy, pegylated liposomal doxorubicin, and oral VP16 [2]. However, the effective rate of the recommended regimens is only 15–28%, and the survival results and PFS/OS are low. Now, in our study, the results firstly showed that ORR was 42%, DCR was 84%, PFS was 9.5 months, and OS was 16.2 months for platinum-resistant recurrent EOC to be received ATO-based sequential combination chemotherapy protocol, which may be new chemotherapy mode.

In the past study [11], a prospective trial evaluated the efficacy of weekly paclitaxel therapy in patients with platinum-resistant EOC. The results showed that the remission rate of patients with platinum-resistant EOC was about 21%, 46% of patients were diagnosed with stable disease, the median response duration was 3.6 months, and the PFS time was 7 months. Furthermore, pegylated liposomal doxorubicin (PLD) and topotecan were used for the treatment of relapsed EOC after being treated with a platinum-containing regimen [12, 13]. The results were compared with topotecan therapy and indicated that ORR to both regimens was remarkably similar (20% vs 17%). The time to progression (TTP) (22 weeks vs 20 weeks) and median OS (66 weeks vs 56 weeks) were also comparable. TRINOVA-1 study reported that the median PFS of trebananib combined with paclitaxel was 7.2 months, while that of the paclitaxel control group was lower (5.4 months) [14]. The median PFS of platinum-resistant EOC patients treated with bevacizumab plus chemotherapy (pegylated liposomal doxorubicin, weekly paclitaxel, or topotecan) in the AURELIA study was 6.7 months, while the median PFS of the chemotherapy alone was 3.4 months [15]. The CARTAXHYII clinical study showed that the median PFS of paclitaxel plus topotecan was 5.4 months and the median PFS of carboplatin and paclitaxel group was 4.8 months, which is a better outcome compared with the median PFS of a single-dose paclitaxel (3.7 months) [16]. The combination of apatinib with oral etoposide in the AEROC study shows promising efficacy with a median PFS of 8.1 months in 33 patients with platinum-resistant or platinum-refractory EOC [17]. These studies demonstrated that the median PFS of recurrent drug-resistant EOC patients was short and there was no effective treatment plan. Compared their regimens, we firstly designed a chemotherapy protocol of ATO-based sequential combination chemotherapy protocol in the study, which is a good clinical effect and may be a new chemotherapy protocol for drug-resistant EOC patients.

### 4.2. The Unique Antitumor Effect and Broad-Spectrum Activity of Arsenic Trioxide (ATO)

Several studies have shown that ATO is an effective agent in the treatment of hematological malignancies. Consequently, the U.S. Food and Drug Administration (FDA) approved the combination of ATO and all-trans retinoic acid (ATRA) for the treatment of acute promyelocytic leukemia (APL) in 1995. Guo Wei et al. used ATO combined with VP16 and paclitaxel to treat metastatic osteosarcoma and Ewing sarcoma in China [4]. After two courses of chemotherapy, the results shown that 15.6% (5/32) were completely relieved, 18.8% (6/32) were partially relieved, 40.6% (13/32) had stable disease, and 25% progressed (8/32). The results showed that ATO combined with other chemotherapy agents has a beneficial effect on solid tumors. Other studies have also shown that ATO is effective for esophageal, gastric, liver, and other tumors. However, there was little reported about ATO in the clinical treatment of gynecological tumors.

In our study, ATO-based combined sequential chemotherapy was used to treat refractory and recurrent EOC. At the end of follow-up period (median 22.1 months), the results showed that ORR was 42%, DCR was 84%, and PFS was 9.5 months, which demonstrated reliable antitumor effects in resistant EOC patients. It is the first report on the ATO efficacy as a treatment of drug-resistant EOC in China. Our data suggest that ATO could be used for the treatment of drug-resistant and relapsed EOC. However, this is a pilot study, which requires further testing in a larger set of EOC patients.

### 4.3. Mechanism of ATO-Based Combined Chemotherapy for Drug-Resistant and Relapsed Ovarian Cancers

This study shows that ATO combined with sequential chemotherapy is an effective regimen, which could be used for the treatment of recurrent drug-resistant EOC. What potential molecular mechanisms of ATO anticancer effects?

There were ATO-related studies that indicated antitumor effects of this drug in ovarian cancer cells *in vitro* [18]. Zhang et al. showed that ATO may inhibit peritoneal infiltration of ovarian cancer cells *in vivo* and *in vitro* in a dose-dependent manner. ATO may reduce various tumor cell activities, inhibit cell attachment to peritoneal mesothelial cells, enhance the interaction between tumor cells, downregulate the expression level of matrix metalloproteinase (MMP)-2 and MMP-9, and upregulate the expression level of MMP inhibitor (TIMP) [19]. Luo et al. [20] and Smith et al. [21] have shown that ATO could reduce angiogenesis, which may be linked to ATO's effect on the expression levels of VEGF, VEGF receptor 2, transforming growth factor (TGF)-*β*RII, and CD31.

However, the mechanisms of ATO effects on platinum-resistant ovarian tumor cells were incompletely addressed. Kong et al. [22] showed that As_2_O_3_ could induce Fas-dependent apoptosis and S phase blockade in cisplatin-resistant ovarian cancer cell lines. Yuan et al. [23] found that ATO induced apoptosis in cisplatin-resistant ovarian cancer cells via reduction of phosphorylated AKT (p-AKT) levels and activation of caspase-3 and caspase-9. Kodigepalli et al. [24] and others found that ATO could inhibit the growth of ovarian cancer cisplatin-resistant cell line COC1/DDP. The effect may be associated with ATO-induced apoptosis in ovarian cancer drug-resistant cells. The inhibition of ovarian cancer cell growth was also linked to upregulation of tumor suppressor genes (Bax, p53, etc.) and downregulation of the expression of lung drug-resistant protein (LRP) *in vitro*. ATO was also shown to inhibit the growth of ovarian cancer cisplatin-resistant cell lines transplanted in the abdominal cavity of nude mice in *vivo*. The effect may be mediated by Fas, nm23H1, N-Myc, and MTA1 gene regulation [24].

Now, it has been shown that ATO combined with other tumor chemotherapy drugs provokes synergistic antitumor effects in drug-resistant ovarian cancer cells. Zhang et al. [25] used a combination index (CI) analysis to show that As_2_O_3_ and cisplatin have synergistic antiproliferation and proapoptotic effects in ovarian cancer cells and cisplatin-resistant cancer cells. Dose reduction index (DRI) data analysis also showed chemotherapy dose reduction by ATO. Byun et al. [26] found that As_2_O_3_ and As_4_O_6_ could inhibit the growth and stimulate apoptosis in paclitaxel-sensitive and drug-resistant ovarian cancer cell lines. In conclusion, As_2_O_3_ and/or combined with other chemotherapeutic drugs, such as paclitaxel, may exert proapoptotic effects in platinum-resistant ovarian cancer cells via activation of various anticancer mechanisms. Therefore, ATO-based combined chemotherapy regimens may be representing clinical benefits in the treatment of platinum-resistant EOC.

### 4.4. The Clinical Significance of ATO-Based Combined Sequential Chemotherapy for Drug-Resistant Relapsed and Refractory Advanced EOC

The sequential chemotherapy method was developed according to the Norton–Simon hypothesis. The definition means that the non-cross-resistant drugs should be used sequentially; for instance, an appropriate dose of chemotherapy protocol A is administered for several cycles and followed by a relevant dose of protocol B for several cycles. Thus, a combination of different mechanisms of chemotherapy drugs/regimens could be used to target the heterogeneous tumor cells, which may allow to increase the effectiveness of chemotherapy and reduce the drug-related adverse reactions. Theoretically, sequential chemotherapy helps to overcome the intratumor heterogeneity and may be a better treatment strategy. Sequential chemotherapy has been used in the treatment of high-risk trophoblastic tumors, breast cancer, and lung cancer, which demonstrated better effectiveness compared with conventional combined chemotherapy regimens. Li et al. [27] described a clinical study with 41 EOC patients, and the results found that sequential chemotherapy improves the PFS and reduces the side effects compared with the conventional chemotherapy protocol. The findings suggest that sequential chemotherapy may improve the survival and life quality of patients with EOC.

In this study, Regimen 1 and Regimen 2 were designed to test the effects of ATO-based combined chemotherapy. The results showed that these regimens have a better ORR and DCR and demonstrate good beneficial effects for patients with primary and secondary drug-resistant EOC after first-line and multiline treatments. It was also shown could prolong the platinum-free interval (PFI) for patients with platinum-resistant EOC. In this study, for 5 cases with drug-resistant EOC patients adopting regimen 2, after PFI more than 12 months during the chemotherapy period, they were also sequentially treated with oxaliplatin. The results have shown that after platinum-free protocol chemotherapy prolonged PFI for more than 12 months. The results showed that the sequentially contained oxaliplatin regimen could improve the prognosis of resistant platinum EOC. However, the small number of cancer cases is the limitation of our study. Therefore, a larger sample study is required to confirm our findings.

The main side effects of ATO-based sequential combined chemotherapy included myelosuppression (100%), minor gastrointestinal and liver toxicities, and skin toxic reactions. The grade 3 and more main included myelosuppression were reversed to normal after treatment with granulocyte-stimulating factor. The toxic side effects are relatively controllable, which indicates good tolerance to the regimen in drug-resistant cancer patients after multicourse or multiline chemotherapy.

This study is a single-center investigation, which represents another limitation of this research. Future studies should use expanded patient samples and be conducted under multicenter investigation protocols such as randomized, blinded, and controlled clinical testing.

## Figures and Tables

**Table 1:** Patient characteristics, all patients enrolled (*n* = 33), and the International Federation of Gynecology and Obstetrics (FIGO).

FIGO stage	No.	%
IC	3	9%
IIA	2	6%
IIB	1	3%
IIIA	1	3%
IIIB	3	9%
IIIC	14	42%
IV	9	27%
Histology at diagnosis		
High-grade serous carcinoma	26	79%
Low-grade serous carcinoma	1	3%
Mixed carcinoma (serous and endometrioid)	1	3%
Clear cell carcinoma	5	15%

**Table 2:** Study drug exposure.

Courses of chemotherapy	No.	%
3	1	3%
4	5	15%
5	2	6%
6	6	18%
7	9	27%
8	2	6%
9	3	9%
10	1	3%
11	2	6%
12	2	6%

Scheme	No	%
1	6	18%
2	27	82%

**Table 3:** Treatment responses.

Index	Total no.	Scheme 1	Scheme 2
No. of patients	33	6	27
Complete response (CR)	6 (18%)	1 (17%)	5 (19%)
Partial response (PR)	8 (24%)	1 (17%)	7 (26%)
Stable disease (SD)	14 (42%)	3 (50%)	11 (41%)
Progressive disease (PD)	5 (15%)	1 (17%)	4 (15%)
Objective response rate (ORR)	14 (42%)	2 (33%)	12 (44%)
Disease control rate (DCR)	28 (84%)	5 (83%)	23 (85%)

**Table 4:** Possible treatment-related adverse events in the safety population.

Adverse event	Any grade		Grades 3 to 4	
Any adverse event	No	%	No	%
Marrow suppression	33	100%	24	73%
Anemia	28	85%	4	12%
Neutropenia	33	100%	23	70%
Thrombocytopenia	12	36%	1	3%
Drug-induced liver injury	6	18%	0	0
Pyrexia	6	18%	0	0
Diarrhea	2	6%	0	0
Nausea	1	3%	0	0
Vomiting	1	3%	0	0
Abdominal pain	1	3%	0	0
Constipation	1	3%	0	0
Abdominal distension	1	3%	0	0
Mucositis/stomatitis	1	3%	0	0
Abdominal pain, upper	1	3%	0	0
Peripheral neuropathy	1	3%	0	0
Rash	1	3%	0	0

## Data Availability

The data used to support the findings of this study are available from the corresponding author upon request.
